# Drug-Drug-Induced Akathisia: Two Case Reports

**DOI:** 10.1155/2020/9649483

**Published:** 2020-04-23

**Authors:** Grace Owusu Aboagye, Daniel Ankrah

**Affiliations:** ^1^Department of Psychiatry, Korle Bu Teaching Hospital, P. O. Box KB 77, Accra, Ghana; ^2^Department of Pharmacy, Korle Bu Teaching Hospital, P. O. Box KB 77, Accra, Ghana

## Abstract

Extrapyramidal side effects of psychotropic medicines are usually experienced by patients in the first few weeks of initiating therapy. Patients stabilized on these medications who present with distressing complaints akin to akathisia may be triggered by other factors. This report presents two cases of drug-drug-induced akathisia. Case A is a patient with schizophrenia who was being managed with risperidone 2 mg tablet daily for the past 3 years. She fell ill and reported to a nearby clinic where she was prescribed ciprofloxacin and artemether/lumefantrine tablets for the treatment of an infection and malaria. She presented 7 days later to her psychiatrist with complaints of restlessness, tremor, palpitations, insomnia, and resurgence of obsessive thoughts. Case B is a patient who was diagnosed with first-episode psychotic depression and admitted for 10 days. Her medications on admission were fluphenazine decanoate 25 mg depot injection once, olanzapine 10 mg tablet daily, and fluoxetine 20 mg capsule daily. On discharge, ciprofloxacin 500 mg tablet every 12 hours for 5 days and fluconazole 150 mg capsule once were added to her medications for the treatment of a urinary tract infection. She reported back to the hospital a day after discharge with complaints of restlessness, “seizures,” tremor, abdominal discomfort, and weight gain. Both patients were diagnosed with akathisia using ICD-10 classification and the Barnes akathisia rating scale and managed with anticholinergics, benzodiazepines, and beta blockers. Other measures employed in managing the akathisia included reducing the dose of the antipsychotic and/or switching antipsychotics. Despite these management measures, the symptoms of akathisia persisted and only resolved after 4weeks. Upon the resolution of symptoms, Case A continued treatment on olanzapine 5 mg tablet daily and fluoxetine 20 mg capsule daily while Case B continued treatment on risperidone 2 mg tablet daily and fluoxetine 20 mg capsule daily. Using Naranjo's adverse drug reaction causality assessment scale, Medscape drug interaction checker, and literature review, a possible and probable case of drug-drug-induced akathisia was made for Case A and Case B. This report is to create more awareness about psychotropic-antimicrobial-induced akathisia. The information underpins the need for health professionals to consider adverse drug-drug interactions as the probable cause of extrapyramidal side effects experienced by patients on antipsychotics.

## 1. Introduction

There is an increased probability of adverse drug-drug interactions when managing comorbid conditions. Some medications can alter the pharmacokinetics of other medicines when administered together by inducing or inhibiting their metabolism [1, 2]. Adverse effects are commonly reported when drugs such as antibiotics, antiepileptics, antipsychotics, antidepressants, antimalarials, and nonsteroidal anti-inflammatory drugs (NSAIDs) are administered together [1]. One of the adverse effects is akathisia. Akathisia is defined by the Diagnostic and Statistical Manual of Mental Disorders, Fifth Edition (DSM-V), as a condition that causes an inner feeling of restlessness and an urgent need to move. It is commonly associated with, but not limited to, the use of antipsychotics [3–5]. It occurs, in most instances, within four weeks of initiating antipsychotic therapy [6]. Akathisia is diagnosed based on subjective and objective symptoms. Subjective symptoms include restlessness, inner tension, anxiety, panic, irritability, discomfort, and sleeplessness [5, 7]. In severe cases, the thought processes of affected patients may become disorganized and their judgment impaired. Some may also exhibit impulsive behavior and/or have suicidal ideation [7]. Objective symptoms are mainly seen in the movement of patients and can be classified as repetitive, purposeful, stereotypical, or suppressible. Examples include crossing and uncrossing of the legs, pacing, rubbing the scalp or interior thighs, and rocking while sitting. Movements may be accompanied by vocalization such as grunting and moaning [5]. Disease states that commonly cause akathisia are renal impairment, diabetes mellitus, hyperthyroidism, iron deficiency anaemia, Parkinson's disease, and peripheral neuropathy [8]. Akathisia is diagnosed using an assessment tool like the Barnes akathisia rating scale (BARS). Strategies to consider in the management of akathisia are dose reduction or substitution with a drug with lesser propensity to cause akathisia [9]. Augmentation with lipophilic beta blockers (e.g., propranolol) and benzodiazepines may be required. Anticholinergics may also be considered if a patient shows signs of Parkinsonism [6].

Akathisia can lead to treatment nonadherence, a poor prognosis, and an increased risk of suicide if not well managed. Unfortunately, it is often not recognized or misdiagnosed. A more deliberate attempt to identify it using clinical judgment, patient medication history, and assessment tools is encouraged [4]. This report presents two cases of drug-drug-induced akathisia. The purpose is to raise awareness of other possible causes of extrapyramidal side effects presented by patients on psychotropic medications. This will help in the prevention of adverse effects and its negative consequences.

## 2. Case Reports

### 2.1. Case A

**Figure 1:**
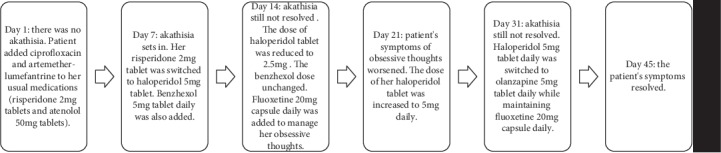
A description of the temporal association between Case A's presentation, treatment, and resolution of akathisia.

**Figure 2:**
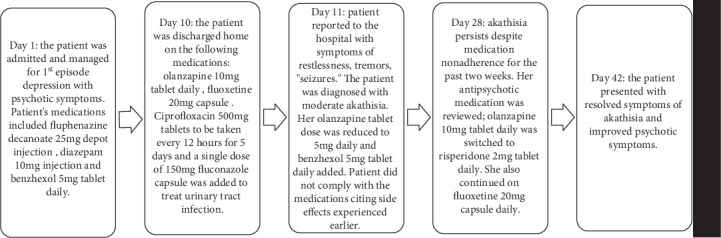
A description of the temporal association between Case B's presentation, treatment, and resolution of akathisia.

Case A was a 54-year-old African female diagnosed with paranoid schizophrenia, according to ICD-10, 3 years ago, and stabilized on risperidone 2 mg tablets daily. On direct questioning by the pharmacist, the patient disclosed visiting a nearby clinic a week ago when she fell ill. The medicines prescribed for her were artemether/lumefantrine and ciprofloxacin tablets. She presented to her psychiatrist a week after her visit to the clinic (day 7) complaining of restlessness, insomnia, feeling tired in the morning, and persistent obsessive thoughts. She had been adherent on her risperidone 2 mg tablet. The patient was also hypertensive and managed uneventfully on amlodipine 10 mg tablet daily, bendroflumethiazide 2.5 mg tablet daily, and atenolol 50 mg tablet daily. She did not drink alcohol or use herbal preparations and had no history of substance abuse. She also had no history of iron deficiency anaemia, diabetes mellitus, or renal failure that could predispose her to restless leg syndrome. There were no abnormal laboratory findings. Physical and mental state examinations ruled out catatonia. She was diagnosed with severe akathisia using ICD-10 classification and a BARS score of 6. Her medications were reviewed; risperidone 2 mg tablet daily was switched to haloperidol 5 mg tablet daily and benzhexol 5 mg tablet daily. The patient continued to take atenolol 50 mg tablet daily. The patient presented for review on day 14 with no improvement in her initial presenting complaints. In view of this, the dose of haloperidol tablet was reduced to 2.5 mg daily with plans to stop gradually. Fluoxetine 20 mg capsule was added to her treatment to be taken daily for the management of her complaints of persistent obsessive thoughts. However, the dose of benzhexol tablet remained unchanged (see Figures 1 and 2 for clarity). The patient presented for review on day 21 with complaints of worsening obsessive thoughts. A decision was taken to increase the dose of the haloperidol tablet prescribed from 2.5 mg daily to 5 mg daily. On day 31, she reported for review with complaints of restlessness and frequent urges to pace around. Her haloperidol 5 mg tablet was switched to olanzapine 5 mg tablet daily while her fluoxetine dosage was maintained. By day 45, when the patient presented for her next review, her symptoms of akathisia had resolved. Using the patient's score of +4 on the Naranjo's adverse drug reaction causality assessment tool [10] (Table 1), the Medscape drug interaction checker, and literature review, a possible cause of the akathisia was attributed to adverse drug-drug interactions between risperidone, ciprofloxacin, fluoxetine, and artemether/lumefantrine.

### 2.2. Case B

Case B was a 17-year-old African female admitted and managed for first-episode depression with psychotic symptoms. Her medications on admission were fluphenazine decanoate 25 mg depot injection, diazepam 10 mg injection once (and thereafter when needed but not to exceed 30 mg in a day), olanzapine 10 mg tablet daily, and benzhexol 5 mg tablet daily when necessary. The patient complained of drooling and psychomotor retardation on admission which resolved when benzhexol 5 mg tablet daily was administered. The patient had no other significant medical history or substance use history and did not consume alcohol or herbal preparations. Her laboratory results on admission showed a reduction in Hb (9.5 g/dl) and a raised platelet count (379 × 109/l). The patient was discharged 10 days after admission. Her discharge medications were olanzapine 10 mg tablet daily, fluoxetine 20 mg capsules daily, a five-day course of ciprofloxacin 500 mg every 12 hours, and fluconazole 150 mg once. The latter two medications were prescribed to treat a urinary tract infection. The patient called her pharmacist a day after discharge (day 11) with complaints of restlessness, tremor, constipation, and “seizures.” She reported back to the hospital the next day and was diagnosed with moderate akathisia using ICD-10 classification and a BARS score of 5. The akathisia was managed by reducing her olanzapine 10 mg tablet to 5 mg daily and adding benzhexol 5 mg tablet daily (to be taken when needed) to her treatment regimen. The patient did not take the prescribed medications on account of the side effects she was experiencing. However, the symptoms of akathisia did not resolve despite noncompliance with her medications. She presented for review on day 28 with complaints of persisting distressing restlessness, weight gain, and stomach upset. Her antipsychotic medication was switched from olanzapine 5 mg tablet daily to risperidone 2 mg tablet daily. At her next review which was on day 42, there were no complaints; neither symptoms of akathisia nor depression with psychosis was present. Using the patient's score of +5 on the Naranjo's adverse drug reaction causality assessment tool (Table 1), the Medscape drug interaction checker, and literature review, a probable cause of the akathisia was attributed to adverse drug-drug interactions between olanzapine, fluphenazine decanoate, ciprofloxacin, and fluconazole. Nevertheless, her low Hb (9.5 g/dl) is a risk factor for akathisia and may have contributed to her developing it.

## 3. Discussion

In the field of psychiatry, antipsychotics are the class of psychotropic medicines usually implicated in akathisia [3, 5], although other medications can cause it [11–14]. Measures taken to address this adverse effect include (1) stopping the medication, (2) reducing the dose, or (3) switching to another antipsychotic and (4) the addition of medications such as anticholinergic agents, benzodiazepines, or propranolol to counteract it [6, 9]. Commonly, extrapyramidal side effects caused by antipsychotics respond to these management measures and patients are observed to be improving with each passing day. However, when these complaints persist over a period of 2 weeks to 1 month and/or have the appearance of a relapse, especially in patients on maintenance therapy, an underlying cause ought to be established. One such underlying cause could be an adverse drug-drug interaction between antipsychotics, antidepressants, and antimicrobials. In 1998, Lane presented a report on the propensity of selective serotonin reuptake inhibitors (SSRIs) to induce akathisia when combined with antipsychotics. In one case report, ciprofloxacin was linked to a probable cause of akathisia in a woman who had taken ciprofloxacin one month earlier for sinusitis prior to presentation of severe akathisia [12]. A case report of agomelatine-duloxetine drug-drug-induced akathisia is also reported in literature [11]. A review of the side effect profiles of the medicines prescribed for Case A and Case B shows that they can all cause symptoms of akathisia [13–17].

Case A presented with complaints of restlessness, tremor, palpitation, insomnia, and resurgence of obsessive thoughts. The obsessive thoughts used to be one of her complaints before being managed and stabilized on risperidone 2 mg tablet daily, and therefore, the obsessive thoughts could be a symptom of a relapse. However, it can also be a symptom of akathisia. Her symptoms of akathisia are consistent with the adverse effects likely to be experienced from a drug-drug interaction between ciprofloxacin, risperidone, fluoxetine, and artemether/lumefantrine. Fluoxetine can increase the plasma levels of risperidone by inhibiting its metabolism by hepatic enzyme CYP2D6. Fluoxetine and risperidone synergistically can also increase the QTc interval. Avoidance of this combination (risperidone+fluoxetine) is recommended. Also, artemether/lumefantrine can increase the level or effect of risperidone by affecting its hepatic enzyme CYP2D6 metabolism. This means two of the medications prescribed (artemether/lumefantrine and fluoxetine) possibly increased the plasma levels of risperidone and slowed its excretion from the patient's body. High blood levels of antipsychotics are associated with akathisia. Furthermore, all the 4 medicines mentioned above can cause akathisia on their own. For example, ciprofloxacin can cause akathisia weeks after completing treatment, even though its elimination rate is 4 hours [12]. In addition, ciprofloxacin-artemether/lumefantrine and ciprofloxacin-fluoxetine interactions have the potential to cause prolongation of the QTc interval. This may result in palpitations and arrhythmias [13]. A similar explanation can be given to the experience of Case B.

Case B's presenting complaints were restlessness, tremor, abdominal discomfort, constipation, and weight gain. These complaints were distressing enough to account for her medication nonadherence. The association between Case B's presenting complaints and the administration of high-dose antipsychotics has been reported in literature [4]. Thus, it may be inferred that the two antipsychotics, olanzapine 10 mg tablet daily and fluphenazine decanoate 25 mg depot once, which constitute a 150% antipsychotic dose in total, contributed to the side effects experienced by Case B. However, the patient did not present with moderate akathisia when on admission and receiving these two antipsychotics, although she presented with drooling and psychomotor retardation which resolved when benzhexol 5 mg tablet daily was administered. Case B's presentation of moderate akathisia may be associated with fluoxetine-fluphenazine interaction and/or olanzapine-ciprofloxacin interaction. It is recommended that the combination of fluoxetine and fluphenazine be avoided as the former inhibits the metabolism of the latter by liver enzyme CYP2D6. Ciprofloxacin also increases the plasma levels of olanzapine by inhibiting its metabolism by CYP1A2 [13]. Also, ciprofloxacin and fluoxetine have akathisia listed as one of their side effects [12–14]. Persisting high plasma concentrations of these medications may have accounted for the development of the akathisia and the long duration in resolution of the symptoms, even when the patient was not adherent to her medications. A similar presentation after a one month of ciprofloxacin administration has been reported in literature [12].

In summary, the key points supporting the assertion that Case A and Case B's akathisia is caused by an adverse drug-drug interaction (and not just extrapyramidal side effects of the individual agents involved) are stated as follows: the scores of Case A and Case B on Naranjo's adverse drug reaction causality assessment scale, the pharmacodynamics and pharmacokinetic properties of the medicines prescribed, the late appearance of the akathisia and its severity, timing of the addition of the ciprofloxacin, and the more than four weeks it took for the akathisia to remit despite two weeks of nonadherence (in the case of Case B) and nonresponse to mitigating measures(in the case of Case A). In addition, both patients recovered and continued on antipsychotics in the same class as the antipsychotics they previously were taking, albeit different molecules, minus the antibiotics and antimalarial.

## 4. Conclusion

Patients may visit other hospitals without disclosing it to their clinicians and neither will they inform them on other medications they are taking if not prompted. Psychotropic-antimicrobial-induced akathisia may be common but underreported or unidentified. This report creates more awareness of its possibility. It will help in the prevention of such adverse effects and its negative consequences on medication adherence and health care costs.

## 5. Recommendation

It is important to conduct an in-depth medication review before a patient is discharged home after hospital admission and at each review. This will aid in preventing and/or identifying other possible and potential causes of extrapyramidal side effects patients on antipsychotic medications may present with. Better still, a facility may go a step further in making the above stated activity part of a bundled care or service to patients with mental disorders.

In patients with mental disorders, a second-generation cephalosporin such as cefuroxime may be considered for the management of the urinary tract infection [18] instead of ciprofloxacin. In the management of the fungal infections and malaria, the prescriber's discretion in stopping the antipsychotic and/or antidepressants or reducing the dose(s) may be required in view of the elimination rates of the medications and their potential for drug-drug interactions.

## Figures and Tables

**Table 1:** Result of Naranjo Adverse Drug Reaction Probability Scale for Case A and Case B.

Question	Yes	No	Do not know	Case A score	Case B score
(1) Are there previous conclusive reports on this reaction?	+1	0	0	+1	+1
(2) Did the adverse event appear after the suspected drug was administered?	+2	-1	0	+2	+2
(3) Did the adverse reaction improve when the drug was discontinued or a specific antagonist was administered?	+1	0	0	+1^∗^^a^	+1^∗^^a^
(4) Did the adverse event reappear when the drug was readministered?	+2	-1	0	Not rechallenged	Not rechallenged
(5) Are there alternative causes (other than the drug) that could on their own have caused the reaction?	-1	+2	0	-1 (relapse)	0
(6) Did the reaction reappear when a placebo was given?	-1	+1	0	Not done	Not done^∗^^b^
(7) Was the drug detected in the blood (or other fluids) in concentrations known to be toxic?	+1	0	0	Not done	
(8) Was the reaction more severe when the dose was increased or less severe when the dose was decreased?	+1	0	0	0	0
(9) Did the patient have a similar reaction to the same or similar drugs in any previous exposure?	+1	0	0	0	0
(10) Was the adverse event confirmed by any objective evidence?	+1	0	0	+1	+1

Total score				+4	+5
